# Adh enhances *Actinobacillus pleuropneumoniae* pathogenicity by binding to OR5M11 and activating p38 which induces apoptosis of PAMs and IL-8 release

**DOI:** 10.1038/srep24058

**Published:** 2016-04-05

**Authors:** Lei Wang, Wanhai Qin, Jing Zhang, Chuntong Bao, Hu Zhang, Yanyi Che, Changjiang Sun, Jingmin Gu, Xin Feng, Chongtao Du, Wenyu Han, Paul Langford Richard, Liancheng Lei

**Affiliations:** 1 College of Veterinary Medicine, JiLin University, Changchun, P. R. China; 2 College of Animal Science, Henan Institute of Science and Technology, Xinxiang, P. R. China; 3 Changchun University of Chinese Medicine, Changchun, P. R. China; 4 Section of Paediatrics, Imperial College London, London, UK

## Abstract

Members of the Trimeric Autotransporter Adhesin (TAA) family play a crucial role in the adhesion of Gram-negative pathogens to host cells, but the immunopathogenesis of TAAs remains unknown. Our previous studies demonstrated that Adh from *Actinobacillus pleuropneumoniae* (*A. pleuropneumoniae*) is required for full bacterial pathogenicity. Alveolar macrophages are the first line of defense against respiratory infections. This study compared the interactions between porcine alveolar macrophages (PAMs) and wild-type *A. pleuropneumoniae* (5b WT) or an *Adh*-deletion strain (5b Δ*Adh*) via gene microarray, immunoprecipitation and other technologies. We found that Adh was shown to interact with the PAMs membrane protein OR5M11, an olfactory receptor, resulting in the high-level secretion of IL-8 by activation of p38 MAPK signaling pathway. Subsequently, PAMs apoptosis via the activation of the Fax and Bax signaling pathways was observed, followed by activation of caspases 8, 9, and 3. The immunological pathogenic roles of Adh were also confirmed in both murine and piglets infectious models *in vivo*. These results identify a novel immunological strategy for TAAs to boost the pathogenicity of *A. pleuropneumoniae*. Together, these datas reveal the high versatility of the Adh protein as a virulence factor and provide novel insight into the immunological pathogenic role of TAAs.

Adhesion is a key factor in the colonization and pathogenesis of *A. pleuropneumoniae.* Trimeric autotransporter adhesin (TAA) is an important virulence factor in Gram-negative bacteria/host cell interactions^1^. TAAs mainly mediate bacterial adhesion, biofilm formation, auto-agglutination and the early bacterial pathogenic process^2^. A variety of gram-negative pathogenic bacteria possess TAAs, such as YadA on the surface *Yersinia enterocolitica* (the first discovered TAA)^3^, NadA on *Neisseria meningitidis meningitis*^4^, UpaG on uropathogenic *Escherichia coli*^5^, BadA on *Bartonella henselae*^6^, BtaE on *Brucella*^7^, and HadA on the cell surface of *Haemophilus influenzae*^8^. Likewise, our laboratory previously confirmed that *A. pleuropneumoniae* also possesses a TAA, named Apa1, and further confirmed via gene deletion that the 68–612 aa head domain, named Adh, is the main functional domain of Apa1^9^.

*A. pleuropneumoniae* is the causative agent of contact-contagious pleural pneumonia, which is a typical respiratory disease characterized by hemorrhagic and necrotizing lung injury^10^. The disease is widely spread worldwide and has caused serious economic losses to the pig industry^11^. *A. pleuropneumoniae* infects the lung specifically. Primary alveolar macrophages (PAMs) are the earliest immune cells that interact with pathogenic microbes during lung infection, and they are an important indicator of the host’s innate and specific immunity. PAMs have multiple biological functions; they can not only take up and present antigens but also regulate the innate inflammatory reaction via the secretion of various molecules, including interleukins, growth factors, prostaglandin, complements, tumor necrosis factor, etc.^12^,^13^. Therefore, exploring pathogen/PAM interactions is necessary to understand the pathogenicity of respiratory infectious microbes.

We have confirmed that Adh is closely involved in bacterial virulence, but its mechanism of action remains unknown. Recent results suggest that an excessive inflammatory immune response is important in the pathogenesis of pneumonia; therefore, we speculated that Adh is likely to induce an excessive immune response that contributes to the pathogenesis of APP, rather than just mediating bacterial adhesion. This study focused on Adh, the functional domain of Apa. From a comparison of the different outcomes of *Adh*-knockout (5bΔ*Adh*) and wild-type (5b WT) strains in the infection of piglet porcine primary alveolar macrophages, mice and piglets, we found that Adh not only mediates bacterial adhesion to host cells but also induces PAMs to undergo apoptosis and release IL-8, which results in the recruitment of excessive inflammatory cells to the lungs. We further confirmed that Adh mediates these activities by interacting with the PAM surface olfactory receptor OR5M11 to induce the phosphorylation of p38 MAP kinase and activate Fas and Bax. This study is the first to confirm that Adh mediates bacterial virulence by interacting with OR5M11, thus illuminating a new pathogenic mechanism for TAA and providing novel targets for bacterial vaccine development and drug treatment.

## Results

### *A. pleuropneumoniae* infection induces PAM apoptosis and the release of inflammatory cytokines

**Figure 1:**
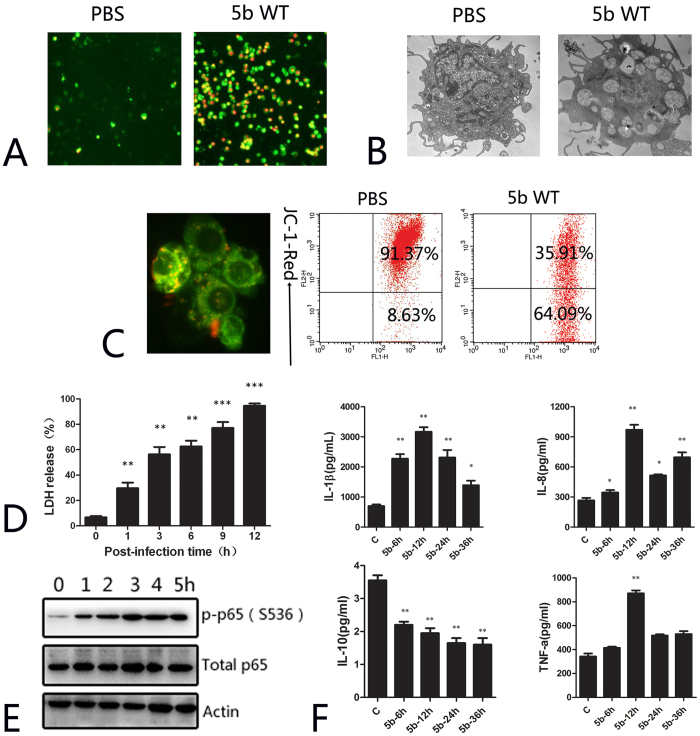
*A. pleuropneumoniae* WT infection induces PAM apoptosis and the release of inflammatory cytokines *in vitro*. (**A**) Annexin V/PI staining of *A. pleuropneumoniae* WT-infected PAMs; (**B**) Observation of *A. pleuropneumoniae* WT-infected PAMs by transmission electron microscopy; (**C**) Flow cytometry results and fluorescence staining of *A. pleuropneumoniae* WT-infected PAMs; (**D**) LDH release of *A. pleuropneumoniae* WT-infected PAMs; (**E**) WB results of the activation of the p65 submit; (**F**) The release of cytokines by *A. pleuropneumoniae* WT-infected PAMs.

Annexin V surface display is the hallmark of early apoptosis^14^. Fluorescence staining for Annexin V indicated that infection with *A. pleuropneumoniae* increased the number of fluorescence-positive cells (Fig. 1A). Transmission electron microcopy (TEM) results showed that the cytoplasm was condensed and deeply stained with various vacuoles, the nucleus was enlarged, the edge of the nucleus was deeply stained, and unevenly distributed, typical apoptotic bodies were apparent around the cell edge of the *A. pleuropneumoniae*-infected PAMs (Fig. 1B). Detection of the mitochondrial membrane potential indicated that *A. pleuropneumoniae* infection resulted in the depolymerization of mitochondrial JC-1. Flow cytometry showed that *A. pleuropneumoniae* infection changed the mitochondrial membrane potential of 64.09% of the cells, a significantly higher result than that obtained with PBS treatment (Fig. 1C). All these results indicate that *A. pleuropneumoniae* infection leads to the apoptosis of PAMs. The release of LDH reflects the degree of bacterial cytotoxicity to the cells. The results indicated that *A. pleuropneumoniae* is cytotoxic to PAMs in a time-dependent manner (Fig. 1D). Activation of the p65 subunit of NF-kB is closely related to the secretion of cytokines^15^. Accordingly, the western blot results showed the activation of NF-kB subunit p65 in PAMs, which peaked 3 h after *A. pleuropneumoniae* infection (Fig. 1E). Cytokine detection in the supernatant of the *A. pleuropneumoniae*-infected PAMs indicated that *A. pleuropneumoniae* infection dramatically increased the release of inflammatory cytokines (IL-1β, IL-8 and TNF-α) but depressed the expression of the anti-inflammatory cytokine IL-10 (Fig. 1F). Taken together, these results indicate that *A. pleuropneumoniae* infection can induce PAM apoptosis and inflammatory cytokine release *in vitro*.

### Adh deficiency significantly reduces PAM apoptosis and cytokine release

**Figure 2:**
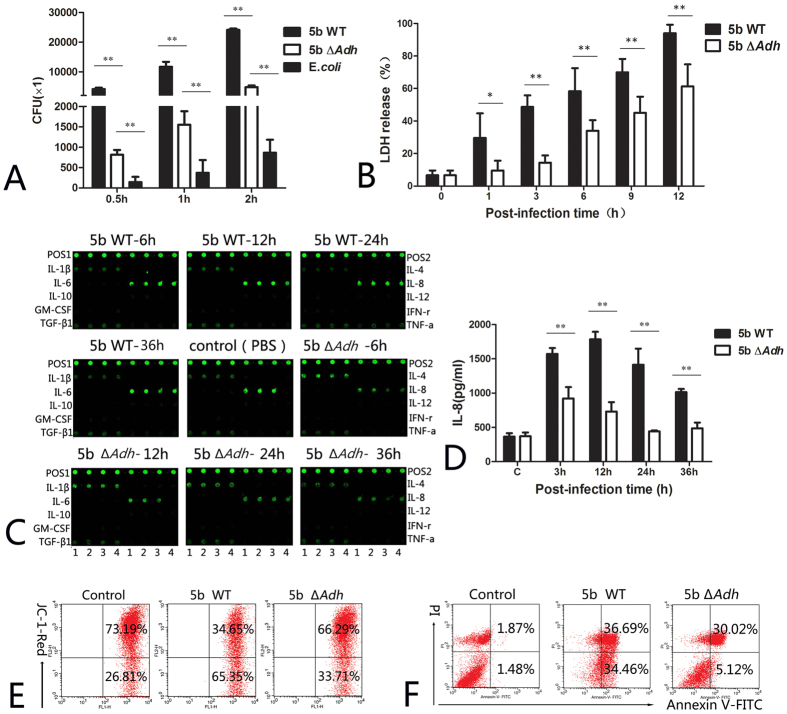
Adh deficiency significantly reduces PAM apoptosis and cytokine release. (**A**) The adhesion of *A. pleuropneumoniae* 5b WT, 5b Δ*Adh* and *E. coli* to PAMs; (**B**) The detection of LDH released by PAMs; (**C**) Cytokine array of *A. pleuropneumoniae* 5b WT- and 5b ΔAdh-infected PAMs; Note: Left side names showed cytokine dots in the left 4 columns each chip, while right side names showed the right 4 columns part in the same chip. (**D**) ELISA results of IL-8 in the supernatants of *A. pleuropneumoniae* 5b WT- or 5b Δ*Adh*-infected PAMs; (**E**) JC-1 staining of *A. pleuropneumoniae* 5b WT- or 5b Δ*Adh-*infected PAMs; (**F**) Annexin V/PI of *A. pleuropneumoniae* 5b WT- or 5b Δ*Adh-*infected PAMs.

TAA primarily mediates bacterial adhesion to host cells^16^. The adhesion and invasion assay showed that the adhesion ability of 5b Δ*Adh* strain was obviously decreased towards PAMs at 0.5 h, 1 h and 2 h , compared with 5b strain, but it was still stronger than that of the control strain (*E. coli*) (Fig. 2A); While Adh deletion also decreased the invasion ability of *A. pleuropneumoniae* towards PAMs (Supplementary Fig 1). *A. pleuropneumoniae* 5b Δ*Adh* infection significant decreased the release of LDH compared with infection with *A. pleuropneumoniae* 5b WT in different times (p < 0.01, Fig. 2B), indicating that Adh contributes to *A. pleuropneumoniae* cytotoxicity toward PAMs. Adh deficiency significantly decreased the capacity of *A. pleuropneumoniae* to change the status of JC-1; in particular, 5b Δ*Adh* infection altered the mitochondrial membrane potential of fewer cells compared with *A. pleuropneumoniae* WT infection (Fig. 2E). The total apoptotic ratio also significantly decreased from 71.15% to 35.14%; the early phase apoptotic ratio decreased much more significantly, from 34.46% to only 5.12% (Fig. 2F), indicating that Adh deficiency resulted in decreased apoptosis. To comprehensively understand how Adh mediated the release of inflammatory cytokines, 10 types of inflammation-related cytokines were measured by array in the supernatant of *A. pleuropneumoniae* 5b WT- or 5b Δ*Adh*-infected PAMs. The release of IL-8 was significantly different between the two strains (p < 0.01, Fig. 2C). ELISA results indicated that IL-8 release from the *A. pleuropneumoniae* 5b WT-infected group was significantly higher than from the *A. pleuropneumoniae* 5b Δ*Adh*-infected group at 3, 12, 24, and 36 h post-infection (p < 0.01, Fig. 2D). IL-8 is an important neutrophil chemokine, and excessive release can aggravate lung inflammation^17^. We therefore speculated that Adh may aggravate pulmonary inflammation by recruiting excessive neutrophils into the lung. These results indicate that Adh mainly induces apoptosis and IL-8 release during the *A. pleuropneumoniae* infection process.

### Adh induces apoptosis in PAMs via Fas activation

**Figure 3:**
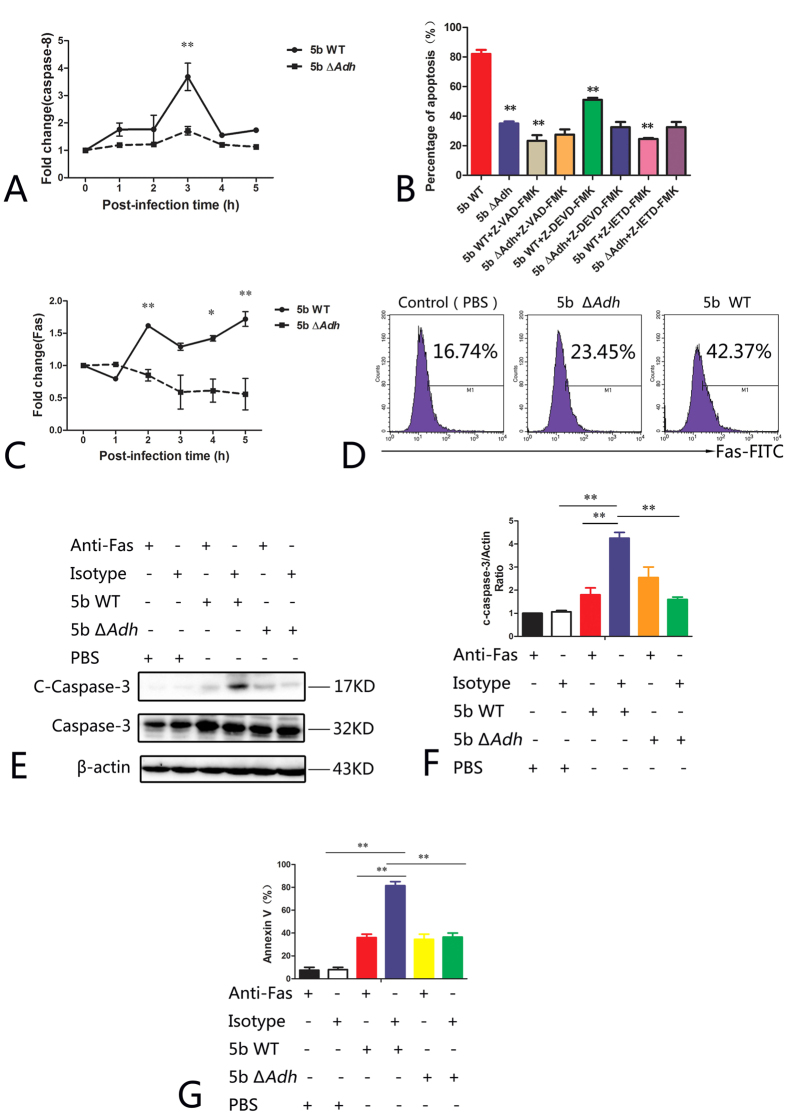
Adh-induce apoptosis of PAMs via the activation of Fas. (**A**) Expression of caspase-8 after *A. pleuropneumoniae* 5b WT or 5b Δ*Adh* infection; (**B**) The effects of caspase inhibitors on 5b WT- or 5b Δ*Adh*-induced apoptosis; (**C**) qRT-PCR identification results of Fas in 5b WT- or 5b Δ*Adh*-infected PAMs; (**D**) Flow cytometry identification results of the expression of Fas in 5b WT- or 5b Δ*Adh*-infected PAMs; (**E,F**) The effects of Fas antibody pretreatment on the activation of caspase-3; (**G**) Flow cytometry results of PAM apoptosis.

Exogenous apoptosis is mediated by the Fas/FasL signaling pathway and caspase-8, which possesses a role in apoptosis signal cascade amplification^18^. In this study, the expression of caspase-8 was significantly higher in 5b WT-infected PAMs than in 5b Δ*Adh-*infected PAMs, and the expression of caspase-8 peaked 3 h post-infection (p < 0.05, Fig. 3A). A caspase inhibition study showed that the caspase inhibitor Z-VAD-FMK and the caspase-3 inhibitor Z-DEVD-FMK significantly inhibited 5b WT-mediated apoptosis but not 5b Δ*Adh*-mediated apoptosis (p < 0.01, Fig. 3B), indicating that Adh is involved in the activation of caspase-8. To further determine whether Adh mediates the activation of Fas, we measured the expression of Fas after infection with *A. pleuropneumoniae*. 5b WT upregulated Fas; in contrast, 5b Δ*Adh* infection slightly downregulated Fas (Fig. 3C). As measured with flow cytometry, the ratio of Fas-positive cells was significantly higher in 5b WT-infected PAMs than in 5b Δ*Adh*-infected PAMs (Fig. 3D). Pretreatment with a Fas-specific antibody significantly inhibited the activation of caspase-3 in 5b WT-infected PAMs but had no significant inhibitory effect on the activation of caspase-3 in 5b Δ*Adh*-infected PAMs (Fig. 3E,F). Annexin V/PI staining revealed that Fas antibody pretreatment significantly inhibited 5b WT-induced apoptosis (p < 0.01) but had no significant effect on apoptosis in 5b Δ*Adh*-infected PAMs (Fig. 3G).

### Adh is involved in the transformation of mitochondrial membrane potentials and the expression of caspase-9

**Figure 4:**
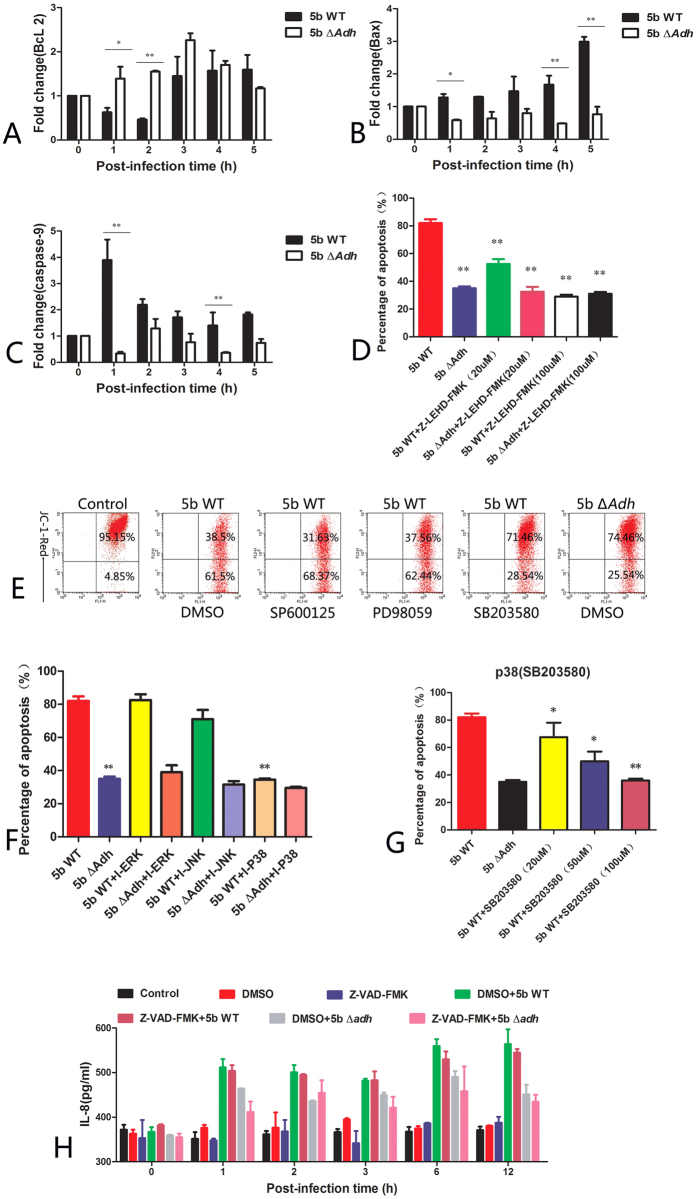
Adh is involved in the transformation of mitochondrial membrane potential and the expression of caspase-9. (**A**) qRT-PCR results of Bcl2 in *A. pleuropneumoniae* 5b WT- or 5b Δ*Adh-*infected PAMs; (**B**) qRT-PCR results of Bax in *A. pleuropneumoniae* 5b WT- or 5b Δ*Adh-*infected PAMs; (**C**) qRT-PCR results of caspase-9 in *A. pleuropneumoniae* 5b WT- or 5b Δ*Adh-*infected PAMs; (**D**) The effects of a Caspase-9 inhibitor on the Adh-mediated apoptosis of PAMs; (**E**) Flow cytometry results of JC-1 in MAPK inhibitor-pretreated PAMs; (**F**) Annexin V/PI staining results of 5b WT- or 5b Δ*Adh-*mediated apoptosis in MAPK inhibitor-pretreated PAMs; (**G**) The effects of different densities of the p38 inhibitor SB203580 on 5b WT-mediated apoptosis; (**H**) The effects of caspase inhibitors on 5b WT- or 5b Δ*Adh*-mediated IL-8 release in PAMs.

*Legionella pneumophila* toxin SidF can inhibit apoptosis via the activation of the Bcl2 family members BNIP3 and BCL-rambo^19^. Bax enhances cell apoptosis, contributing to endogenous apoptosis by the alteration of mitochondrial membrane potentials and inducing the release of cytochrome C to further activate caspases 9 and 3/7 in epithelial cells during *Pseudomonas aeruginosa* infection^20^. Whether Adh can mediate apoptosis via Bcl2 and Bax remains unknown. qRT-PCR results showed that Bcl2 in the 5b WT-infected PAMs was first downregulated and then subsequently upregulated; Bcl2 expression was significantly lower in 5b Δ*Adh*-infected PAMs 2 h post-infection (p < 0.01, Fig. 4A). In contrast, the expression of Bax gradually increased and peaked 5 h post-infection in 5b WT-infected PAMs, whereas expression decreased, rather than increased, in 5b Δ*Adh*-infected PAMs (Fig. 4B). Endogenous apoptosis occurs in a caspase-9 dependent manner^21^. In our study, the expression of caspase-9 was significant in 5b WT-infected PAMs 1 h post-infection, indicating that Adh mediates caspase-9 activation and rapid mitochondrial membrane potential changes in the early phase of *A. pleuropneumoniae* infection (Fig. 4C). To verify this hypothesis, PAM apoptosis was evaluated after a 1-h pretreatment with the caspase-9-specific inhibitor Z-LEHD-FMK before infection with 5b WT or 5b Δ*Adh*. At 100 μM, Z-LEHD-FMK treatment significantly reduced 5b WT-induced apoptosis (p < 0.01, Fig. 4D) but showed no significant effect on 5b Δ*Adh-*mediated apoptosis, which further suggests Adh mediates the activation of caspase-9.

MAP kinase signaling pathways are involved in apoptosis in various types of cells^22^,^23^. Whether the MAP kinase signaling pathways contribute to Adh-mediated apoptosis remains unknown. Pretreatment with MAP kinase signaling pathway inhibitors prior to *A. pleuropneumoniae* 5b WT or 5b Δ*Adh* infection showed that the p38-specific inhibitor SB203580 significantly changed the mitochondrial membrane potential in the PAMs. Annexin V/PI staining results showed that SB203580 also significantly reduced 5b WT-induced apoptosis in a dose-dependent manner (p < 0.01) but had no significant effect on 5b WT-induced apoptosis (Fig. 4F). JNK and ERK inhibitors showed no effect on 5b WT-mediated apoptosis. Evidently, p38 is involved in Adh-mediated apoptosis. However, caspase inhibitors cannot reduce Adh-mediated IL-8 release, suggesting there is no direct relation between Adh-mediated IL-8 secretion and the Adh-mediated apoptosis of PAMs (Fig. 4H).

### Adh mediates IL-8 release through the activation of the MAP kinase signaling pathway

**Figure 5:**
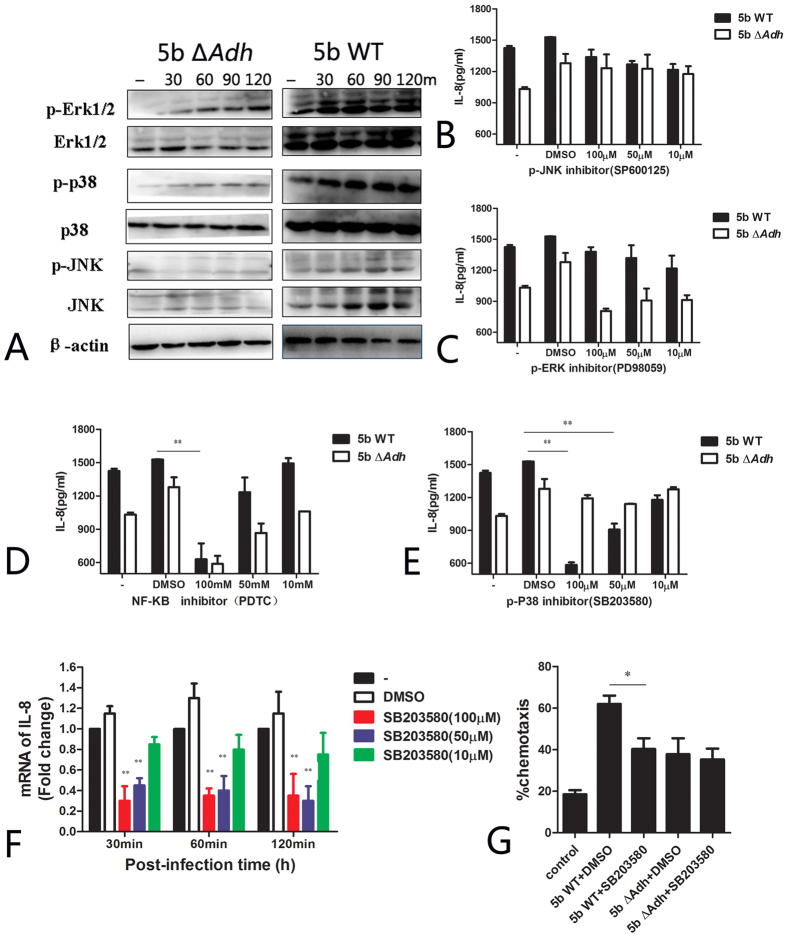
Adh mediates IL-8 release through the activation of the MAPK signaling pathway. (**A**) Activation of MAPK in *A. pleuropneumoniae* 5b WT- or 5b Δ*Adh*-infected PAMs; (**B–E**) The effects of pretreatment with SP600125, PD98059, PDTC, or SB203580 on IL-8 release in *A. pleuropneumoniae* 5b WT- or 5b Δ*Adh*-infected PAMs; (**F**) The effects of pretreatment with SB203580 on the expression of IL-8 mRNA in 5b WT- or 5b Δ*Adh*-infected PAMs; (**G**) The effects of pretreatment with SB203580 on the recruitment of neutrophils in 5b WT- or 5b Δ*Adh*-infected PAMs.

Mitogen activated protein kinases (MAPKs) are involved in multiple biological functions, including cell cytokine secretion, cell differentiation and cell apoptosis^24^. To delineate the mechanisms involved in Adh-mediated IL-8 release, we evaluated the activation of p38, JNK, and ERK by western blotting and found that although *A. pleuropneumoniae* infection can induce the activation of MAPK signaling, 5b WT infection can induce higher levels of phosphorylation of p38 and ERK than 5b Δ*Adh* infection at 30 and 60 min post-infection (Fig. 5A). Pretreatment with the MAPK inhibitors SB203580 and PDTC (which inhibit p38 and NK-kB, respectively) prior to *A. pleuropneumoniae* infection significantly reduced Adh-mediated IL-8 release. Additionally, pretreatment with 100 nM and 50 nM SB203580 extremely significantly and significantly inhibited IL-8 release, respectively (Fig. 5E, p < 0.01), but had little effect on 5b Δ*Adh*-mediated IL-8 release. Therefore, Adh-mediated IL-8 release is closely related to the phosphorylation of p38. Neither ERK nor JNK inhibition exerted inhibitory effects on 5b WT-mediated IL-8 release (Fig. 5B,C). The NF-kB inhibitor PDTC(100 nM) inhibited both mutant and WT *A. pleuropneumoniae* mediated IL-8 release (Fig. 5D), suggesting that LPS and lipoproteins are involved in *A. pleuropneumoniae-*mediated inflammation.

Tyrosine protein kinase is involved in YadA-mediated IL-8 release^25^, although its inhibitor did not inhibit IL-8 release in our study, which indicates that tyrosine protein kinase is not involved in Adh-mediated IL-8 release (data not shown). To further confirm the involvement of p38 phosphorylation in IL-8 release, IL-8 mRNA in *A. pleuropneumoniae*-infected PAMs was collected after treatment with different concentrations of SB203580; p38 inhibition reduced IL-8 expression at different time points (Fig. 5F). IL-8 is a typical neutrophil chemokine that recruits neutrophils to inflammatory tissues^26^. The chemotaxis efficiency of neutrophils toward the supernatants of 5b Δ*Adh*-infected PAMs was much lower than that of 5b WT-infected PAMs (62.5%) (Fig. 5G, p < 0.05). The addition of a p38 inhibitor significantly reduced chemotaxis efficiency (Fig. 5G, p < 0.05), which confirms the involvement of Adh in the *A. pleuropneumoniae*-mediated recruitment of neutrophils. Taken together, p38 phosphorylation is involved in Adh-mediated IL-8 release by PAM and in the recruitment of neutrophils.

### Adh mediates PAM apoptosis and IL-8 release by interacting with OR5M11

**Figure 6:**
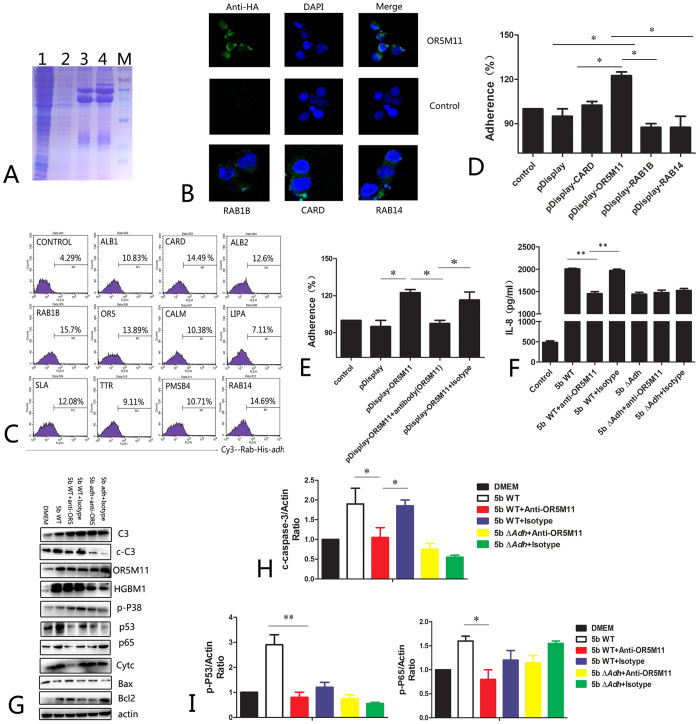
Screening and identification results of PAM surface Adh-interacting proteins. (**A**) Immune co-precipitation results of Adh and the PAM total protein mixture; (**B**) Laser-scanning confocal microscopy results of suspected proteins expressed on the surface of 293 T cells; (**C**) Adh adheres to 293 T cells expressing different proteins; (**D**) *A. pleuropneumoniae* adhered to different protein-expressing 293 T cells; (**E**) *A. pleuropneumoniae* adhered to OR5M11 antibody-pretreated 293 T cells expressing different proteins; (**F**) OR5M11 antibody pretreatment significantly inhibited *A. pleuropneumoniae*-induced IL-8 release by PAMs; (**G,H**) Apoptosis-related proteins expressed in *A. pleuropneumoniae*-infected and OR5M11 antibody-pretreated PAMs; (**I**) p53 activation in *A. pleuropneumoniae*-infected and OR5M11 antibody-pretreated PAMs.

In this study, purified Adh was mixed with PAM total proteins to identify Adh-interacting proteins on the PAM surface via a co-precipitation technique (Fig. 6A). The mass spectrum results yielded 24 proteins that can potentially interact with Adh. Among these proteins, keratin accounts for the highest percent, followed by serum albumin, heat shock proteins, cell adhesion molecules, etc.(Supplementary Table S3); 11 of those 24 proteins were displayed on the surface of 293 T cells mainly based on the protein functions, and their expression rates were estimated using laser-scanning confocal microscopy (Fig. 6B). Flow cytometry showed that CARD, OR5M11, RAB1B and RAB14 were significantly enhanced by Adh adhesion to 293 T cells, and adhesion rates increased more than 3-fold compared with the control group (Fig. 6C). TAAs mediate bacterial adhesion to host cells^16^, and Adh is the key functional domain. OR5M11 expression increased the adherence of *A. pleuropneumoniae* to 293 T cells by 30% (Fig. 6D and Supplement Fig S2, p < 0.05), whereas pretreatment with an OR5M11-specific antibody significantly inhibited *A. pleuropneumoniae* adherence to Adh-expressing 293 T cells (p < 0.05). Similarly, pretreatment of OR5M11 with an OR5M11-specific antibody also significantly inhibited *A. pleuropneumoniae* 5b WT-mediated IL-8 release (p < 0.05, Fig. 6F), whereas an isotype control antibody did not inhibit *A. pleuropneumoniae* adherence (p < 0.05, Fig. 6E). However, the R5M11-specific isotype antibody did not influence 5b Δ*Adh* infection-mediated IL-8 release (p < 0.05, Fig. 6F). Pretreatment with the OR5M11-specific antibody reduced *A. pleuropneumoniae* 5b WT infection-mediated PAM apoptosis (Fig. 6G), as characterized by the significantly reduced activation of caspase-3 (p < 0.05, Fig. 6H) and p53 (p < 0.01, Fig. 6I). In addition, OR5M11-specific antibody pretreatment also inhibited the phosphorylation of p65, which suggests that Adh actives NF-kB to induce IL-8 release by interacting with OR5M11. Taken together, Adh mediates PAM apoptosis and IL-8 release by interacting with OR5M11.

### Adh contributes to *A. pleuropneumoniae* pathogenicity in a mouse model

**Figure 7:**
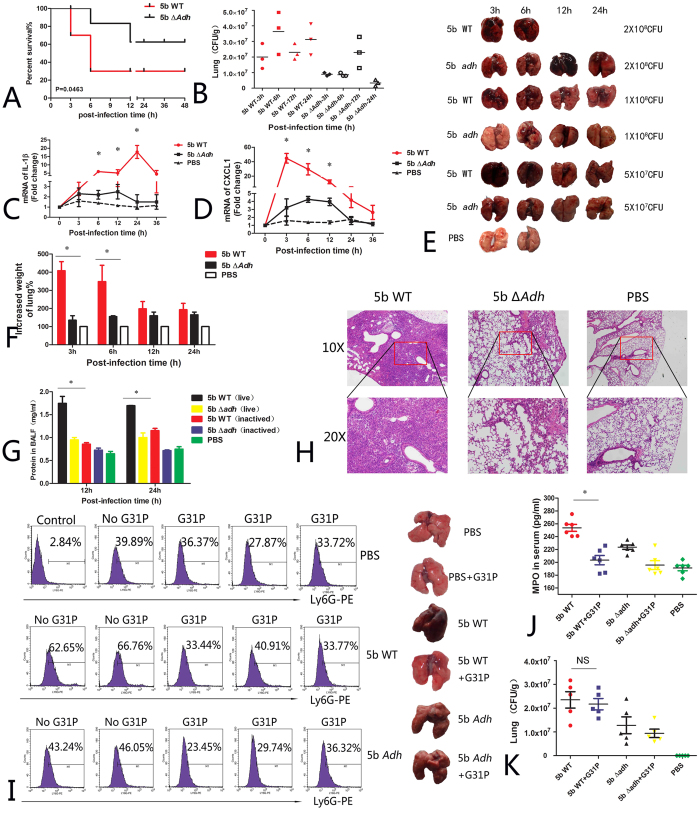
*A. pleuropneumoniae* 5b WT- or 5b Δ*Adh-*intranasally infected BALB/c mice. (**A**) Survival of *A. pleuropneumoniae* 5b WT- or 5b Δ*Adh*-infected mice with an infectious dose of 1 × 10^8^ CFU per mouse; (**B**) bacterial lung burden of *A. pleuropneumoniae*-infected mice; (**C,D**) qRT-PCR results for the expression of inflammatory cytokines in *A. pleuropneumoniae*-infected mice; (**E**) Lung-apparent pathological changes due to different doses of *A. pleuropneumoniae* 5b WT or 5b Δ*Adh* in infected mice; (**F**) Increased lung weights of 5b WT- or 5b Δ*Adh*-infected mice at different time points; (**G**) Total proteins in BALF; (**H**) H&E staining of lungs; (**I**) Neutrophils in the lungs (**I**), MPO in the sera (**J**) and the bacterial lung burden (**K**) of G31P-treated mice.

When mice were infected with *A. pleuropneumoniae* at a dose of 1 × 10^8^ CFU per mouse, the survival rate of *A. pleuropneumoniae* 5b WT-infected mice was significantly higher than that of 5b Δ*Adh-*infected mice (60% vs. 30%, respectively, p = 0.0462, Fig. 7A). The bacterial lung burden was lower in the *Adh*-mutant mice (p < 0.05, Fig. 7B). The expression of proinflammatory cytokine IL-1β and the neutrophil chemokine CXCL1 were higher in 5b WT-infected mice throughout the course of infection (Fig. 7C,D). IL-1β peaked 24 h post-infection, which was 17-fold higher than in 5b Δ*Adh-*infected PAMs and PBS controls (P < 0.05, Fig. 7C). CXCL1 rapidly increased 45-fold 3 h post-infection and then decreased throughout the remainder of the time course, which was in accordance with the development of pleuropneumonia; therefore, CXCL1 might be used as a novel biomarker to test the course of the disease in mice(Fig. 7D). In addition, lung lesions in 5b Δ*Adh*-infected mice were less apparent than those in 5b WT-infected mice at different doses (Fig. 7E); lung weights were significantly lighter than in 5b WT-infected mice at the early phase (6 h) (P < 0.05, Fig. 7F), and total protein in BALF was also lower in 5b Δ*Adh*-infected mice (P < 0.05, Fig. 7G), suggesting that Adh deletion decreases pulmonary congestion and hemorrhaging. H&E staining of the lung sections showed that lung lesions were more apparent in 5b WT-infected mice and exhibited lung alveolar rupture, lung congestion, and the infiltration of a large number of inflammatory cells in the pulmonary interstitium and alveoli and around the bronchia, all of which are typical of bronchial pneumonia. Lung lesions were relatively less apparent in 5b Δ*Adh*-infected mice but also exhibited increased pulmonary interstitium widths; however, the inflammatory cells were mainly concentrated around the bronchus, and fewer inflammatory cells infiltrated the alveoli (Fig. 7H). These results indicate that Adh contributes to *A. pleuropneumoniae* pathogenicity in mice.

IL-8 and CXCL1 are both neutrophil chemokines, and excessive neutrophil accumulation can aggravate lung injury. Previous results showed that Adh was involved in pleuropneumoniae-mediated CXCL1 release in mice, which led us to speculate that CXCL1 was the main factor causing pneumonia. G31P is a chemokine antagonist^27^ that can reduce lung injury caused by *Klebsiella pneumonia*^28^. In this study, pretreatment with G31P significantly relieved *A. pleuropneumoniae-*caused lung lesions, decreased lung congestion, ameliorated lung burden and decreased neutrophil recruitment to the lung (Fig. 7I). Decreased MPO in the blood also suggested that G31P pretreatment significantly decreased the activation of neutrophils (Fig. 7J). However, G31P pretreatment exerted limited effects on lung burden and cytokine release (Fig. 7K).

### Piglet model infection confirms Adh involvement in apoptosis and IL-8 release

**Figure 8:**
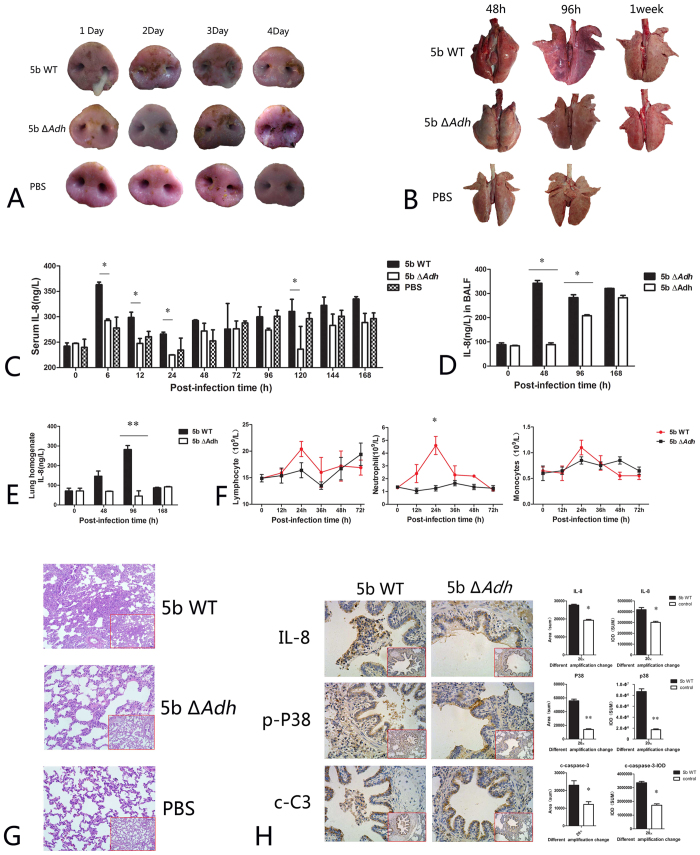
Intranasal infection of *A. pleuropneumoniae* 5b WT or 5b Δ*Adh* in a piglet model. (**A**) Clinical symptoms of *A. pleuropneumoniae* 5b WT- or 5b Δ*Adh*-infected piglets; (**B**) Pathological changes in the lungs of infected piglets; (**C–E**) IL-8 level in the sera (**C**), BALF (**D**) and lung homogenates (**E,F**) Peripheral blood routine test results of infected piglets; (**G**) H&E staining results of the lungs (HE); (**H**) Immunohistochemical results of IL-8, phosphorylated p38 and activated caspase-3 in the infected lungs.

In a piglet model, depression and other typical early clinical symptoms of infectious pleuropneumonia appeared earlier in the 5b WT-infected group, high fevers appeared 12 h post-infection with body temperatures rising to more than 40 °C, and anorexia appeared 24 h post-infection with purulent nasal discharge (Fig. 8A). Comprehensive scores were also higher in the early phase^9^. Piglets were sacrificed at 48 h, 96 h, and 1 week post-infection to estimate pathological lung changes. There were significant differences in the lung lesions of piglets between the 5b WT- and 5b Δ*Adh*-infected groups; the lung lesions were much more apparent in *A. pleuropneumoniae* 5b WT-infected mice, and large areas of congestion and necrosis were observed, especially in the early infection phase (Fig. 8B). In addition, Adh played a key role in *A. pleuropneumoniae* colonization of the lung, blood and bronchoalveolar lavage fluid (BALF) (Supplementary Fig 3). The IL-8 serum levels in 5b WT-infected piglets were significant higher than in 5b Δ*Adh*-infected piglets at 6, 12, 24, 48, 72, 96, 120, 144 and 168 h post-infection (p < 0.05, Fig. 8C), which is consistent with the *in vitro* results. IL-8 in the BALFs and lung homogenates of 5b WT-infected piglets were also significantly higher than those of 5b Δ*Adh*-infected piglets to some degree (p < 0.05 or p < 0.01, Fig. 8D,E). A routine blood test showed significantly more neutrophils in the peripheral blood in 5b WT-infected piglets 12 and 24 h post-infection (p < 0.05, Fig. 8F). Lymphocytes were also more numerous 24 h post-infection (p < 0.05, Fig. 8F), whereas mononuclear macrophages showed no difference (p < 0.05, Fig. 8F). We suspect that increased neutrophils in the peripheral blood are closely related to high levels of IL-8 in the sera, thus promoting neutrophil migration from the bone marrow to the peripheral blood.

H&E staining revealed obvious lung lesions in 5b WT-infected piglets and the typical signs of pneumonia in the lung, which include the thickening and fusion of the alveolar wall, increased pulmonary interstitium widths, and a large number of inflammatory cells (mainly neutrophils and monocytes) recruited to the alveoli, as seen in the mouse model. In contrast, 5b Δ*Adh*-infected piglets exhibited only weak symptoms of pneumonia with smaller lung lesions, less inflammatory cell infiltration into the alveoli, a thin alveolar wall, and no obvious increase in interstitial widths (Fig. 8G). These results suggest that Adh is involved in *A. pleuropneumoniae*-mediated lung lesions in the piglet model, which is consistent with previously reported results^9^. Immunohistochemical results showed that IL-8 release was substantial in the lung, and a large number of inflammatory cells infiltrated the lung trachea. However, the high expression of IL-8 was concentrated in the trachea in *A. pleuropneumoniae* 5b WT-infected piglets. The analytical results showed that IL-8 release was significantly greater in *A. pleuropneumoniae* 5b WT-infected piglets than in *A. pleuropneumoniae* 5b Δ*Adh-*infected piglets (p < 0.05, Fig. 8H). Additionally, the activation of p38 was also significantly higher in *A. pleuropneumoniae* 5b WT-infected piglets (p < 0.05, Fig. 8H). The activation of caspase-3 is a hallmark of apoptosis. In the present study, activated caspase-3 was highly expressed in *A. pleuropneumoniae* 5b WT-infected piglets (p < 0.05, Fig. 8H). In addition, the expression of Bad, Bax, Bcl2, Cytc, caspase-12, p-PI3K, p65, FasL, Fas, caspase-8, and activated caspase-8 were consistent with their counterparts in cellular levels (Supplementary Figs 4 and 5). Taken together, the results of this study confirm that Adh contributes to *A. pleuropneumoniae-*mediated apoptosis and IL-8 release in tissues. In other words, Adh is involved in the early pathogenicity of *A. pleuropneumoniae* in piglets via a novel virulence mechanism.

## Discussion

This study confirmed that Adh contributes to the pathogenicity of *A. pleuropneumoniae* by interacting with OR5M11 to activate the p38 MAPK signaling pathway, resulting in PAM apoptosis and IL-8 release. Adh deletion reduces the interactions between bacteria and PAMs, resulting in lower phosphorylation of p38 that decreases IL-8 release and apoptosis of PAMs, and then decreases the recruitment of neutrophils, finally alleviating the *A. pleuropneumoniae* mediated lung inflammatory lesions. This result not only provides a new target for the prevention and control of porcine contagious pleuropneumonia but also reveals a novel function for TAAs during the infection process.

Adhesion and invasion are key processes during various bacterial infections^29^. Trimeric autotransporter adhesion (TAA) is an important virulence factor that enhances bacterial adhesion to host cells and mediates biofilm formation^30^,^31^. Current studies suggest that excessive inflammation is an important pathogenic mechanism in many diseases. However, whether TAA contributes to bacterially mediated excessive inflammation in the host was previously unknown. YadA was reported to mediate *Yersinia* invasion of epithelial cells and the release of high levels of IL-8 by interacting with components of the membrane surface matrix (ECM), followed by the binding of β-1 ligatin to phosphate adhesion kinase (FAK), Ras activation (which can activate the ERK MAPK signaling pathway), and finally IL-8 release^32^. The present study indicates that Adh interacts with OR5M11 on the PAM surface, resulting in IL-8 release via the activation of the p38 MAPK signaling pathway. OR5M11 belongs to the G protein-coupled receptor (GPCR) family. GPCRs, one of the largest transmembrane receptor families^33^, can respond to extracellular signals and transmit them into cells, enabling them to mediate many physiological functions, such as the cardiovascular system, immune system, nervous system and endocrine system, thus making them important drug targets. The American scientists Robert Lefkowitz and Blaine Kobilka were awarded the Nobel Prize for Chemistry in 2012 for their breakthrough in revealing these protein-coupled receptors and their internal mechanism. IL-8 can interact with CXC subunit receptors to induce cascade effects, resulting in cell damage^34^. Our results indicated that the OR5M11 is a interacting protein with Adh, and involved in the pathopoiesis of *A. pleuropneumoniae* mainly through activation of p38 phosphorylation mediated apoptosis and IL-8 release. Additionally, TAA has not been reported to interact with the G protein-coupled receptor OR5M11 yet; this research enriched the funcetion of OR5M11, and linked OR5M11 with the bacterial infection. Further functional studies will be carried out in the future.

Interleukin-8 (IL-8), a type of neutrophil-activating chemokine^35^, is a base heparin-binding protein that possesses endogenous leukocyte chemotaxis and activation functions^36^. IL-8 can also stimulate eosinophil and T lymphocytes^37^. Various stimuli can induce the production of IL-8. At present, the main substances are microbial lipopolysaccharide (LPS)^38^, cytokines such as tumor necrosis factor α (TNF-α)^39^, granulocyte macrophage-colony stimulating factor (GM-CSF)^40^, interleukin-1β, plant lectin, etc. LPS interacts with TLR4 to activate MyD88, followed by the activation of the NF-kB p65 submit, resulting in the release of inflammatory cytokines such as IL-8^41^. The present study confirms that Adh induces IL-8 release by PAMs and recruits neutrophils. Increasing numbers of studies have suggested that IL-8 is closely related to the development of acute lung injury (ALI) and acute respiratory distress syndrome (ARDS)^42^. IL-8 promotes neutrophil degranulation and the release of elastase and damages endothelial cells, which results in the stasis of blood flow in microcirculation, tissue necrosis and organ dysfunction, thus promoting lung inflammation and injury in the early phase of ALI/ARDS^43^,^44^. The clinical feature of *A. pleuropneumoniae* infection is cellular pleuropneumonia. Adh may play an important role in the development of this disease as a novel virulence factor that induces high levels of IL-8 and the recruitment of neutrophils in the lung, promoting inflammation and injury.

Tissues clear useless cells by means of cellular apoptosis, and blocking this kind of dead cell clearance will prolong inflammation^45^,^46^. Prolonged inflammation will induce the excessive apoptosis of lung epithelial cells, resulting in cellular pneumonia^47^,^48^. Recent studies have shown that the Fas/FasL pathway-mediated apoptosis of alveolar epithelial cells is a potentially important causative factor in acute respiratory distress syndrome (ARDS)^49^,^50^. The expression of Fas and FasL was significantly increased in the lungs of respiratory distress syndrome patients not only in the alveolar epithelial cells, which were infiltrated by inflammatory cells, but also in the caduceus epithelial cells and in the damaged alveolar space^51^,^52^. In our study, the expression of FasL and Fas was significantly higher in *A. pleuropneumoniae* WT-infected piglets, and both genes were also more highly expressed in the WT- and Adh mutant-infected piglets than in the PBS control group. Thus, Adh enhances the *A. pleuropneumoniae*-mediated activation of FasL and Fas. Additionally, the expression level of both genes was elevated in tracheal epithelial cells, indicating that *A. pleuropneumoniae* also induces apparent apoptosis in the tracheal epithelium as well as PAMs, which is consistent with the expression of FasL and Fas in the lungs of typical ARDS patients^51^,^52^. The activation of Fas/FasL promotes the activation of caspase-8^53^. We also observed significantly higher activation of caspase-8 in *A. pleuropneumoniae*-infected piglets than in 5b Δ*Adh*-infected piglets. Increased caspase-8 activation resulted in higher activation levels of caspase-3, the executor of apoptosis, as confirmed by an analysis of lung cell apoptosis.

**Figure 9:**
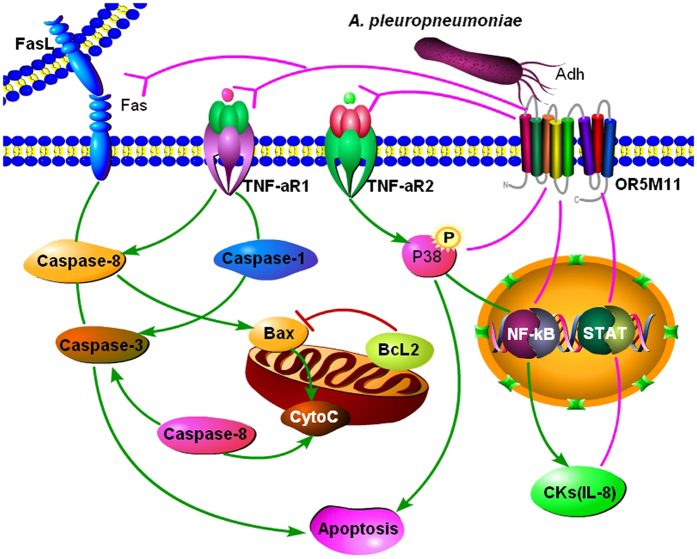
The mechanisms of Adh-mediated interactions between *A. pleuropneumoniae* and PAMs.

Taken together, the results of this study thoroughly outlined the underlying mechanisms that mediate the pathogenicity of *A. pleuropneumoniae* toward PAMs. Our results show that Adh contributes to IL-8 release and to PAM apoptosis via the activation of Fas/FasL, Bax and TNF-aR mainly by interacting with OR5M11 on the PAM surface (Fig. 9). These findings contradict the traditional theory that TAAs only mediate bacterial adhesion and instead expand on their functions and mechanisms, as well as provide new targets for the prevention and control of this type of bacterial disease.

## Materials and Methods

### Ethics Statement

The 6- to 8-week-old BALB/c mice used for bacterial infection were purchased from the Experimental Animal Center of Jilin University and fed according to standardized laboratory protocols. The 45-day-old weaned piglets used for the collection of pulmonary primary macrophages and for bacterial infection were purchased from a pig-breeding farm. All animal experimental procedures were performed in strict accordance with the Regulations for the Administration of Affairs Concerning Experimental Animals approved by the State Council of People’s Republic of China (1988.11.1).

### Bacterial Strains and Cells

*A. pleuropneumoniae* serotype 5 strain L20 (5b WT) was obtained from the Shanghai Entry-Exit Inspection and Quarantine Bureau. The trimeric autotransporter adhesins-deletion strain (5b Δ*Adh*) was constructed and stored by our laboratory. Primary PAMs were collected from the BALF of healthy weaned piglets, which were purchased from a pig-breeding farm. The cells was cultured in DMEM (Gibco, Carlsbad, USA) containing 10% fetal calf serum in 5% CO_2_ at 37 °C.

### The Detection of the Cell Apoptosis Rate

The PAMs were infected at an MOI of 10 and collected at different times (1–5 h) to determine the cell apoptosis rate^54^. Suspended cells were collected directly into an EP tube and centrifuged at 1000 rpm for 5 min. Cell monolayers were disrupted with 0.25% trypsin. Next, the cells were washed once in the PBS buffer and centrifuged at 1,000 rpm for 5 min. The cells were marked with Annexin V/PI (BD, batch no. 556547) and incubated in the dark for 10–15 min at room temperature.

The transformation of color from red to green is indicative of early cell apoptosis^55^. The PAMs were collected after infection with *A. pleuropneumoniae* and stained with 1 ml JC-1 dye (Beyotime, China, JC-1-C2005). The cells were incubated for 20 min at 37 °C and centrifuged at 1,000 rpm for 5 min. The supernatant was discarded, and the precipitate was washed twice with JC-1 staining buffer and incubated on ice. The cells were resuspended in 2 ml cell culture medium and examined by fluorescence microscopy, laser scanning confocal microscopy or flow cytometry.

### Detection of LDH, MPO and CK

The PAMs (1 × 10^5^) were inoculated into a 96-well plate and incubated for 5 h. LDH detection (Roche, batch no. 4744926001) was performed at 3, 6, and 12 h after infection with *A. pleuropneumoniae*. A positive control group was prepared 15 min prior to detection. Cytotoxicity was calculated as follows: %cytotoxicity = (cpmexperimental-cpmspontaneous)/(cpmmaximum-cpmspontaneous) × 100^11^,^56^.

The detection of MPO and cytokines was performed with ELISA using the manufacturer’s manual. Additionally, a cytokine detection chip (Guangzhou Reboo Biological Technology Co. Ltd) was used.

### Realtime quantitative RT-PCR

qRT-PCR was used to verify the cytokine, apoptotic signaling pathway- associated protein. The primer sequences are listed in Supplementary Table S1, 2. RNA extraction and reverse transcription was performed with the TaKaRa RT reagent kit with gDNA Eraser (Perfect Real Time, lot no. RR047A) according to the manufacturer’s instructions.

### Adhesion and Invasion count

PAMs were directly inoculated into a cell plate and used to perform an adhesion experiment after adhering to the plate wall. The medium was changed to penicillin- and streptomycin-free 1640 culture solution, in which the tested strains were added at an MOI of 100. The mixtures were incubated at 37 °C for 30 min, 1 h, or 2 h. The cells were washed with PBS buffer 3 times and then treated with pancreatin. The solutions were diluted 103- to 106-fold with PBS, and 100 μL of the solution were removed and coated onto a BHI culture plate overnight at 37 °C. The number of bacteria was counted to calculate the adhesion rate in three replicates^11^.

The bacterial strains and cells used for invasion were the same used in the adhesion experiment. Furthermore, the dose of infection was the same. *A. pleuropneumoniae* was co-incubated with the PAMs at 37 °C for 30 min, 1 h, or 2 h, The cells were washed with PBS buffer 3 times, treated with gentamycin (at a final concentration of 50 μg/ml) for 1 h, and again washed with PBS buffer three times. Subsequently, the cell lysis solution was added. The solutions were diluted 10^2^- to10^3^-fold with PBS, and 100 μL of each solution was removed and coated onto a BHI culture plate overnight at 37 °C. The number of bacteria was counted to calculate the invasion rate in three replicates^11^.

### Detection of the activation of the signal transduction pathway by Western blot

Pulmonary primary macrophages were inoculated into a 6-well culture plate (2.5 × 10^6^ per well) and infected with *A. pleuropneumoniae* at an MOI of 10. Total protein was extracted after the cells were incubated for 15, 30, 60, 90, and 120 min at 37 °C. The cells were lysed with RIPA (50 mM Tris-HCl, pH 7.4, 150 mM NaCl, 1% NP-40, 0.1% SDS), and the lysate was transferred into a 1.5-ml Eppendorf tube and centrifuged at 14,000 × g for 20 min at 4 °C to collect the supernatant, which was stored at 4 °C.

### Immune co-precipitation

The cells were lysed in RIPA, and then the total proteins (at pH 7.4) were incubated with purified Adh. The Adh-interacting proteins were obtained by immune co-precipitation according to the protocols provided in the Co-Immunoprecipitation (Co-IP) Kit (no. 26149, Pierce). The pulled-down proteins were identified by mass spectrum and classified according to their annotated functions (http://www.uniprot.org/uniprot/).

### Expression and identification of interacting proteins

The coding genes of suspected interacting proteins were cloned into the pDisplay plasmid, and then the reconstructed plasmids were transferred into 293 T cells for surface display. The primers used are listed in Supplementary Table 2. Transfection was conducted with the X-tremeGENE HP DNA Transfection Reagent (Roche, no. 06365752001) according to the manufacturer’s protocol. The expression of target proteins was identified with fluorescence staining. Briefly, the target protein was incubated with a 500-fold diluted anti-HA monoclonal antibody (Genescript) for 30 min with the target, washed twice, and stained with a FITC-conjugated anti-mouse IgG (Life Technologies). Then, the nuclei were stained with Hoechst. Finally, the stained cells were characterized with fluorescence microscopy, laser scanning confocal microscopy and flow cytometry^57^.

### Animal infection

All the animal experiments were performed in accordance with Chinese legislation and all experimental protocols were approved by the independent animal ethics committee at Jilin University.

#### Mice

5b WT and 5b Δ*Adh* strains were cultured to log phase, and washed three times with PBS for challenging experiments via intranasal pathway^[1]^. BALB/c Mice(18~22 g) were divided into 7 groups: two high-dose groups (5b WT and 5b Δ*Adh* strains, 2 × 10^8^CFU /mouse, n = 15/group), two middle dose groups (1 × 10^8^CFU/mouse, n = 15/group) and two low-dose groups (5 × 10^7^CFU/mouse, n = 15/group) and control group (PBS , n = 10). The clinical symptoms were recorded and lung pathological changes (Hematoxylin and Eosin staining), lung bacterial burden, expression of cytokines (Real-time qRT-PCR) and the percentage of neutrophils (flow cytometry). Bronchoalveolar lavage was performed to determining the amount of total proteins in BALF.

For G31P therapy, Mice were divided into 5 groups: two drug treatment groups (5b WT and 5b Δ*Adh* strains + G31P, n = 10/group), two untreated groups (5b WT and 5b Δ*Adh* strains + normal saline, n = 10/group) and the control group (+PBS, n = 5). Mice were challenged intranasally (challenging dose 1 × 10^8^CFU/mouse) and treated with G31P subcutaneously (final concentration 20 μg/kg) 0 h postinfection. The clinical symptoms and other parameters were measured as above.

#### Piglets

Piglets (aged 5 weeks, weighing 10–12 kg,) were randomly divided into three groups: 5b WT and 5b Δ*Adh* (n = 5 each group) and PBS control group (n = 2). Animals were challenged intranasally with 2–3 × 10^8^ CFU (2 mL) bacteria and clinical symptoms were observed and scores were measured^9^.

### Flow-cytometric analysis of lymphocytes in spleens and lungs

*A. pleuropneumoniae*-infected mice were sacrificed, and the lungs and spleens were collected and homogenized on ice. The homogenates were resuspended with PBS. After being washed twice, the cells were incubated with a fluorochrome-conjugated anti-cytokine antibody (APC-anti-CD3, FITC-anti-CD11b, FITC-anti-CD19, PE-anti-F4/80, PE-anti-Ly6G, PE-anti-CD8, or FITC-anti-CD4 antibodies) diluted in PBS for 1 h at room temperature in the dark. The cells were washed twice with PBS for flow cytometric analysis with a FACSC (BD Biosciences), and the data were analyzed with FlowJo (Tree Star Inc.)^58^.

### Immunohistochemistry

The lung tissue was fixed for 3 days in 4% formaldehyde. Next, the tissue was embedded in paraffin and cut into 5-μm sections. After rehydration, the sections were heated in a microwave for antigen retrieval and then sequentially incubated with 3% H_2_O_2_ to block endogenous peroxidase activity and with 3% bovine serum albumin (Sigma-Aldrich) to block nonspecific staining. Primary antibodies, which were diluted 1:100 in 3% bovine serum albumin (BSA), against p38, caspase-3, activated caspase-3, LC3B, Bad, Bax, Bcl2, Cytc, caspase-12, HGBM1, p-PI3K, activated p65 submit, FasL, Fas, caspase-8, and activated caspase-8 were employed at 4 °C overnight in a humidified chamber. All sections were counterstained with hematoxylin and fixed under glass covers with Aquatex^59^. The immunohistochemical staining was examined under a microscope and analyzed using ImagePro Plus 2.0. The differences among groups were analyzed with SPSS19.

### Statistical Analysis

Data analysis and differences (One-Way ANOVA or Two-Way ANOVA) were performed with GraphPad Prism 5. Differences were considered significant when *P < 0.05. Differences were considered extremely significant when ***P < 0.001.

## Additional Information

**How to cite this article**: Wang, L. *et al.* Adh enhances *Actinobacillus pleuropneumoniae* pathogenicity by binding to OR5M11 and activating p38 which induces apoptosis of PAMs and IL-8 release. *Sci. Rep.* **6**, 24058; doi: 10.1038/srep24058 (2016).

## Supplementary Material

Supplementary Information
