# Supplementary material for: Adh enhances Actinobacillus pleuropneumoniae pathogenicity by binding to OR5M11 and activating p38 which induces apoptosis of PAMs and IL-8 release
Source: Sci Rep. 2016 Apr 5;6:24058. doi: 10.1038/srep24058 (PMC4820727; doi:10.1038/srep24058)
Supplement: Supplementary Information [file srep24058-s1.pdf]

Adh enhances *Actinobacillus pleuropneumoniae* pathogenicity by binding to OR5M11 and activating p38 which induces apoptosis of PAMs and IL-8 release

Lei Wang [a, b], Wanhai Qin [a], Jing Zhang[c], Chuntong Bao [a], Hu Zhang [a], Yanyi Che [a], Changjiang Sun [a], Jingmin Gu [a], Xin Feng [a], Chongtao Du [a], Wenyu Han [a], Langford Paul Richard[d], Liancheng Lei [a] *

[a] College of Veterinary Medicine, JiLin University, Changchun, P. R. China

[b] College of Animal Science, Henan Institute of Science and Technology, Xinxiang, P. R. China

[c] Changchun University of Chinese Medicine, Changchun, P. R. China

[d] Section of Paediatrics, Imperial College London, London, UK

Supplementary figure 1

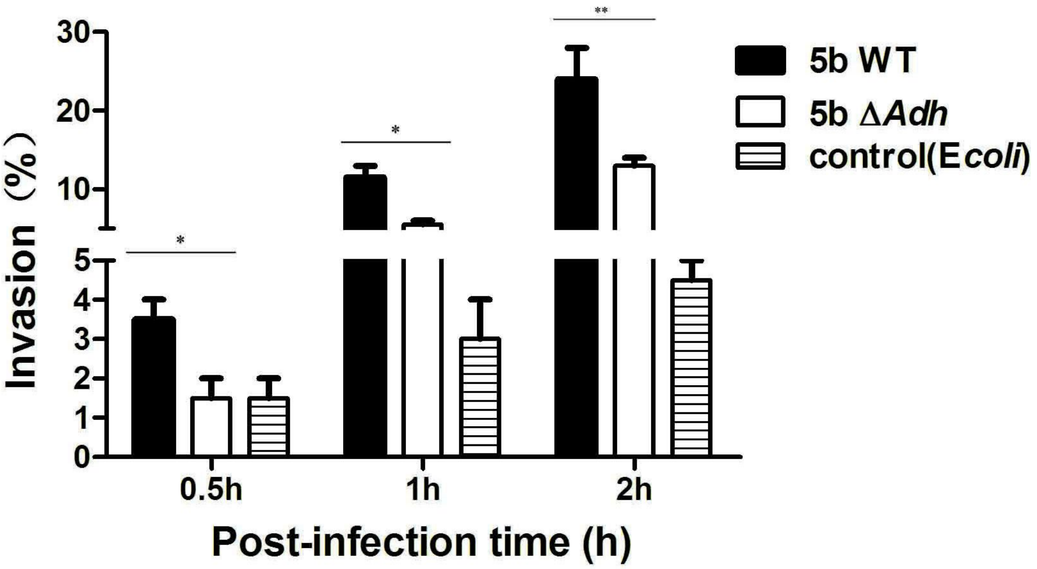

Supplement Fig S1: Invasion of *A. pleuropneumoniae* 5b WT and 5b Δ*Adh* to PAMs.

Supplementary figure 2

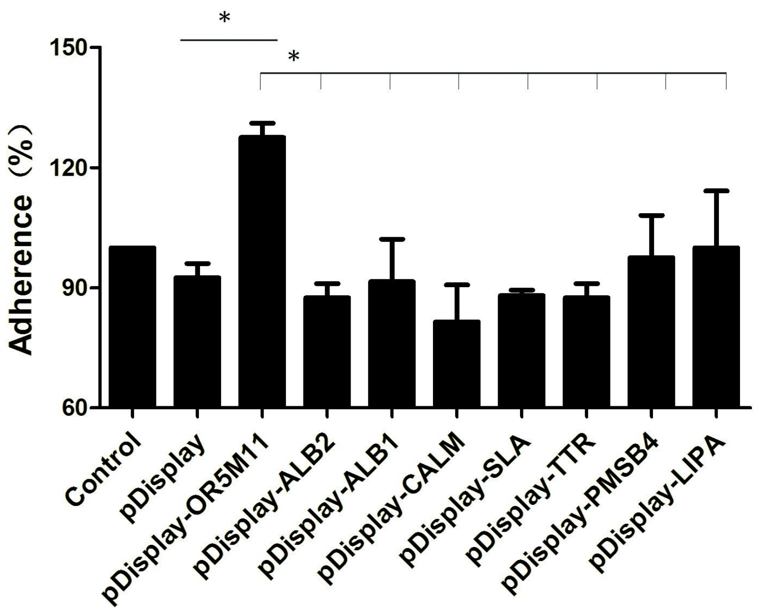

Supplement Fig S2:The expression of ALB2 ALB1 CALM SLA TTR PMSB4 LIPA on the adhesion of *A. pleuropneumoniae*

Supplementary figure 3

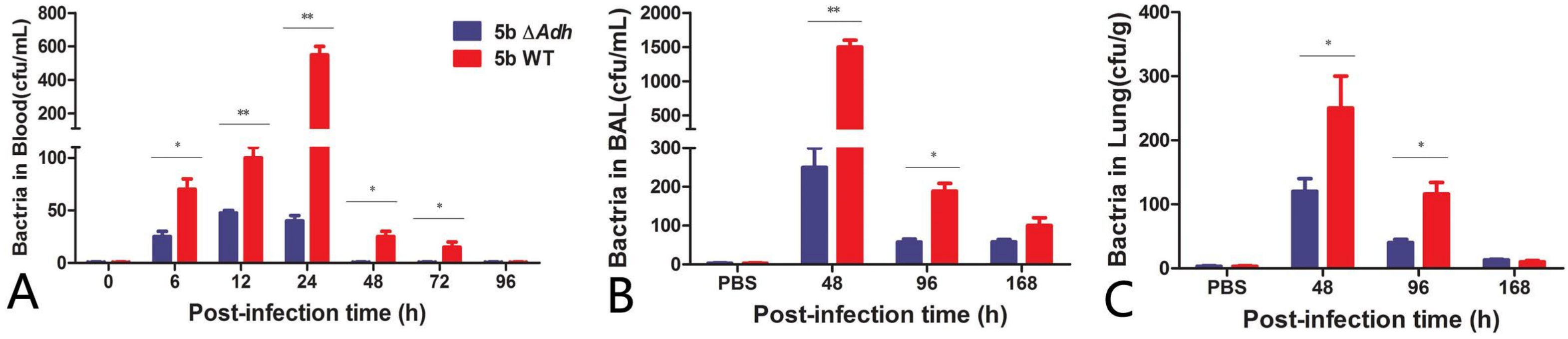

Supplement Fig S3: *A. pleuropneumoniae* colonization in the lung, blood and bronchoalveolar lavage fluid.

Supplementary figure 4

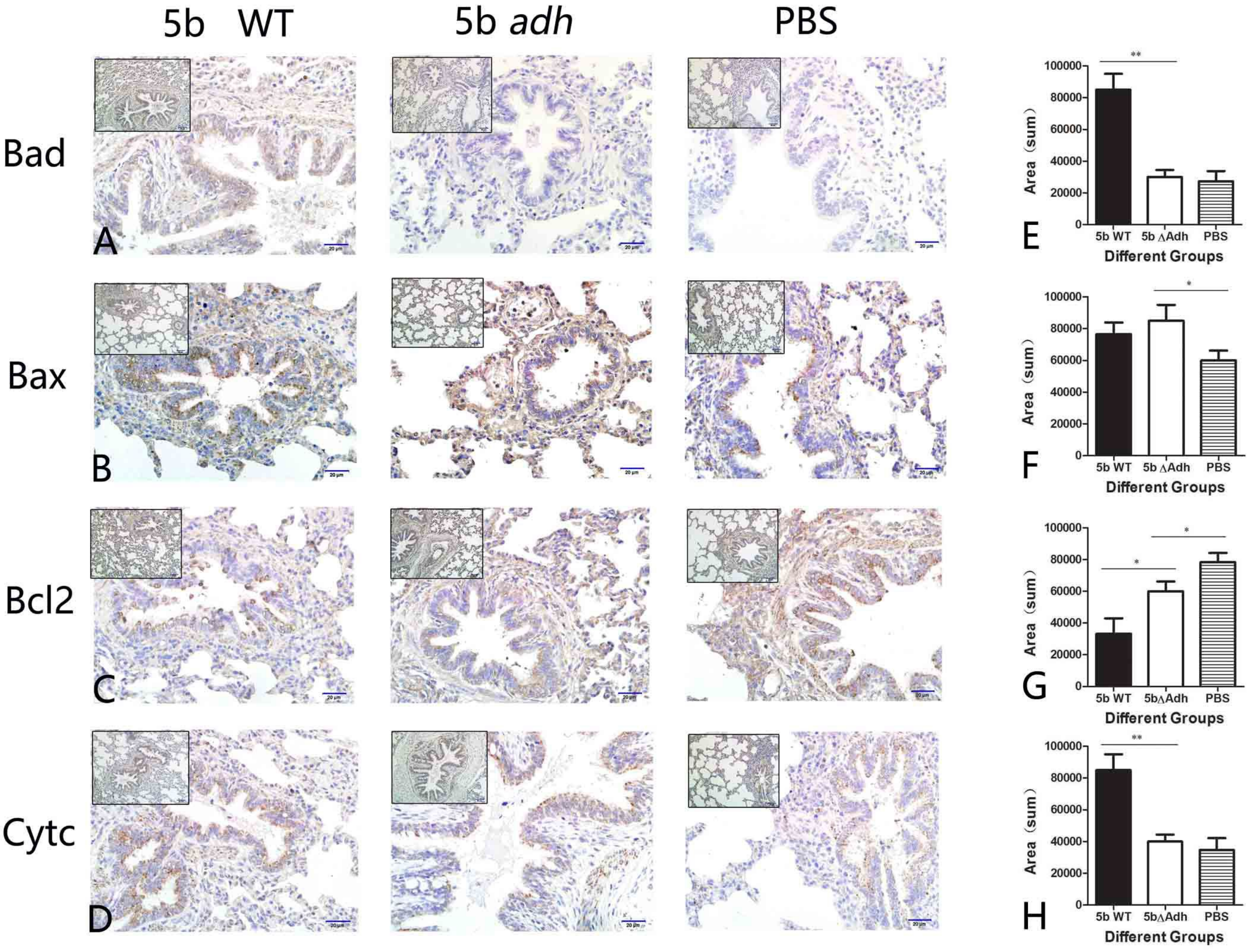

Supplement Fig S4: The expression levels of Bad, Bax, Bcl2 and Cytc in the lungs of piglets infected with *A .pleuropneumoniae*, assessed immunohistochemically.

Supplementary figure 5

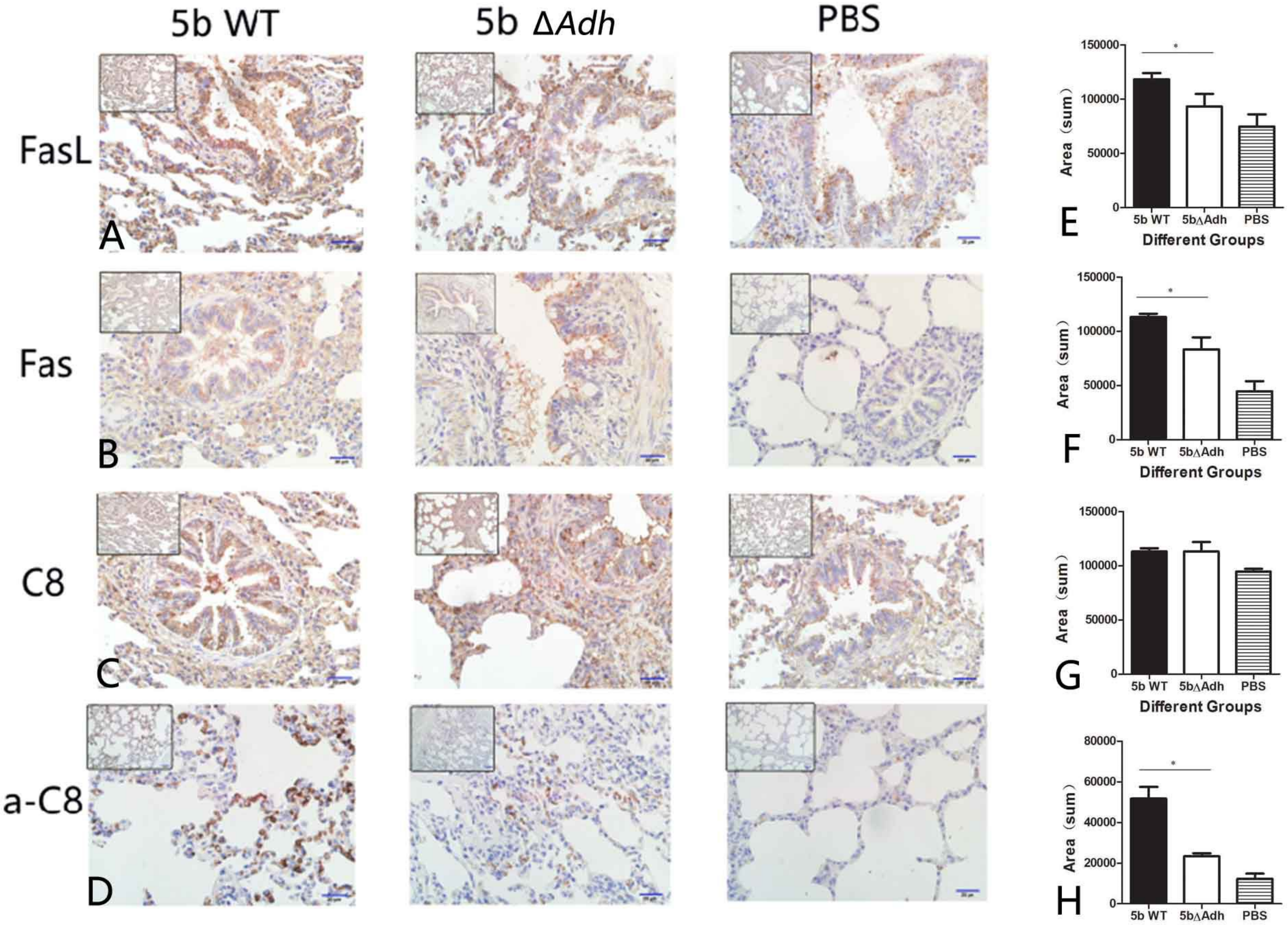

Supplement Fig S5: The expression levels of FasL, Fas, caspase-8, and activated caspase-8 in the lungs of piglets infected with *A. pleuropneumoniae*, assessed immunohistochemically.

Supplementary Table S1: The primers of porcine cytokines and apoptosis related gene for quantitative RT-PCR detection

Genes	Sequence
IL-1β	F: 5'- AGTGGAGAAGCCGATGAAGA -3'<br>R: 5'- CATTGCACGTTTCAAGGATG -3'
IL-6	F: 5'- CCTCTCCGGACAAAACTGAA -3'<br>R: 5'- TCTGCCAGTACCTCCTTGCT -3'
IFN-g	F: 5'- TCAGAGCCACATTGTCTCCTTC-3'<br>R: 5'- CATTCAGTTTCCCAGTGCTACCA-3'
IL-8	F: 5'- TGTGAGGCTGCAGTTCTGGCAAG -3'<br>R: 5'- GGGTGGAAAGGTGTGGAATGCGT -3'
IFN-β	F: 5'-CTCTCCTGATGTGTTTCTCC-3'<br>R: 5'-GTTCATCCTATCTTCGAGGC-3'
TNF-a	F: 5'- CCACGCTCTTCTGCCTACTGC-3'<br>R: 5'- CTCGGCTTTGACATTGGCTAC-3'
IL-10	F:5'-GCTGCGGCGCTGTCATCAATT-3'<br>R:5'- ACCCATGGCTTTGTAGACACCCC -3'
CCL2	F:5'-CAGGTCCTTGCCCAGCCAGATG -3'<br>R:5'- CACAGATCTCCTTGCCCGCGA -3'
CCL4	F:5'-TCCCACCTCCTGCTGCTTCACAT -3'<br>R:5'- GCCTGCCCTTTTTGGTCTGGAA -3'
TNF-aR1	F: 5'- CGCATCGCCGTCTCCTACCA -3'<br>R: 5'- GCCCAGATTCAGCAAAGTCCAGAT -3'
TNF-aR2	F: 5'-GCCTTGCCTTCTATCCTTTATC-3'<br>R: 5'-CGTATCTCCACCAACACCCTAT-3'
caspase-1	F: 5'-GCGTATTCAGAGCCGAGAGGGAG-3'<br>R:5'-CAGATTATGAGGGCAAGGCGTGT-3'
caspase-3	F: 5'-GTGGGATTGAGACGGACAGTGGG-3'<br>R:5'-CGCTGGACAAAGTGACTGGATGA-3'
caspase-8	F: 5'-GAGACAAGGGCATCATCTACGGC-3'<br>R:5'-TGGGTTTACCACGAAGGGAAGG-3'
Fas	F: 5'-TATCGAAGAAACCAAAATAGAC-3'<br>R:5'-CGGAGCAGCTGGACTTTCTG-3'
FasL	F: 5'-CCCATACCCCCAAATCTTCT-3'<br>R:5'-CTGGACAGGGGAAGACTGAG-3'
Caspase-9	F: 5'- CCTTACCCTGCCTTACCTT-3'<br>R:5'- GCTGCCGCATCCTTCA-3'
BAX	F: 5'-CTCAAGCGCATTGGAGATGA-3'<br>R:5'-GTCCACGGCTGCGATCA-3'
BCL-2	F: 5'-CTTTGTCAGGCTTATGAAGGTT-3'<br>R:5'-TGTCCTTTGTCCCATAATAATT-3'
β-catin	F: 5'-CCACCCAGAAGACTGTGGAT-3'<br>R:5'-AAGCAGGGATGATGTTCTGG-3'

Supplementary Table S2：The primers used for qRT-PCR verification of cytokines of mice infected by APP

**Genes**	**Sequence**
IL-1β	F: 5’-GAGCACCTTCTTTTCCTTCATCTT-3’
	R: 5’-TCACACACCAGCAGGTTATCATC-3’
IL-6	F: 5’- TGGATGGTCTTGGTCCTTAGCC-3’
	R: 5’- ACTGATGGTGACAACCACG-3’
IL-12	F: 5’- GTGAACCTCACCTGTGACACGC-3’
	R: 5’- TGAATACTTCTCATAGTCCCTTTGG-3’
CXCL1	F: 5’- CCTGGGATTCACCTCAA-3’
	R: 5’- TTCTGAACCAAGGGAGC -3’
CXCL15	F: 5’- CATCTTCGTCCGTCCCT-3’
	R: 5’- TCCAAACACATCATACTCCC-3’
TNF-a	F: 5’-ATGAGCACAGAAAGCATGATCC-3’
	R: 5’-ACAAGCAGGAATGAGAAGAGG-3’
β-Actin	F: 5’-ATCTACGAGGGCTATGCTCTCC-3’
	R: 5’-CTGATCCACATCTGCTGGAAGG-3’

Supplementary Table S3：The plain appraisal results of target proteins could interact with Adh

Order	Gene Name	Function	CoverPercent	MW
1	KRT79	PIG Uncharacterized protein	14.39%	57900.92
2	ALB	PIG Serum albumin	9.23%	69691.42
3	HSAP2	PIG Uncharacterized protein	12.42%	69821.99
4	IQGAP1	Ras GTPase-activating-like protein	7.10%	149834.58
5	RAB1B	PIG Ras-related protein Rab-1B	23.38%	22111.79
6	IGHG	PIG IgG heavy chain	9.36%	51246.37
7	SLA1	PIG MHC class I antigen	6.78%	33608.09
8	SLA-DRA	PIG MHC class II antigen	6.35%	28425.43
9	RAB14	PIG Actin related protein 2/3 complex subunit 2	3.00%	34278.55
10	C3	PIG Complement C3	0.36%	186804.62
11	APOE	PIG Apolipoprotein E	2.84%	36598.86
12	ITIH4	PIG Inter-alpha-trypsin inhibitor heavy chain H4	1.09%	102144.97
13	CARD	PIG Uncharacterized protein	0.90%	112804.06
14	CALM1	PIG Calmodulin 1	15.58%	8893.76
15	PSMB4	PIG Proteasome subunit	3.79%	29052.77
16	OR5M11	PIG Olfactory receptor	12.18%	36138.69
17	TTR	PIG Transthyretin	4.00%	16081.27
18	FGF7	PIG Fibroblast growth factor 7	5.15%	22462.9
19	CARD4	PIG Caspase recruitment domain member 4	0.91%	62080.92
20	NOD1	PIG Nucleotide-binding oligomerization domain 1	0.52%	107542.33
21	ARHGEF2	PIG Rho guanine nucleotide exchange factor 2	0.73%	108481.85
22	LIPA	PIG Lipase	2.01%	45312.67
23	ODZ4	PIG Ficolin alpha	3.10%	34681.25
24	FCN2	PIG Ficolin-2	0.58%	289365.34
